# Effect of vitamin D supplementation on lower extremity motor function and ambulation in stroke patients

**DOI:** 10.3906/sag-2010-287

**Published:** 2021-06-28

**Authors:** Ayça UTKAN KARASU, Gülçin KAYMAK KARATAŞ

**Affiliations:** 1 Department of Physical Medicine and Rehabilitation, Gazi University Faculty of Medicine, Ankara Turkey

**Keywords:** Stroke rehabilitation, vitamin D, brunnstrom recovery stage, functional ambulation

## Abstract

**Background/aim:**

The aim of this study was to investigate the effect of vitamin D supplementation on ambulation and mobility in hospitalized patients undergoing stroke rehabilitation.

**Materials and methods:**

This study was conducted retrospectively between September 2020 and October 2020 at Gazi University Faculty of Medicine Physical Medicine and Rehabilitation Department. Seventy-six patients who received inpatient stroke rehabilitation treatment between May 2018 and February 2020 were included in the study. The patients were divided into two groups as those who did and did not take vitamin D supplements. Lower extremity motor function and ambulation status were compared using Brunnstrom recovery stage (lower extremity) and functional ambulation classification (FAC) scores before and after rehabilitation.

**Results:**

Thirty-nine patients received vitamin D treatment during the rehabilitation process and 37 patients did not. The two groups were similar in terms of age, sex, time since stroke, stroke type, comorbid diseases, nutritional status, rehabilitation duration, and FAC and Brunnstrom scores before rehabilitation (p > 0.05). At the end of rehabilitation, the changes in FAC and Brunnstrom scores were higher in patients receiving vitamin D supplementation (p = 0.005 and p = 0.018). The change in FAC and Brunnstrom scores in patients who were undergoing rehabilitation for the first time and/or in the first 3 months after stroke was higher in the group receiving vitamin D supplementation compared with the group not receiving vitamin D (p < 0.05). In patients who were not within the first 3 months after stroke, vitamin D treatment did not affect FAC and Brunnstrom scores.

**Conclusion:**

Vitamin D supplementation may increase the success of rehabilitation therapy in patients during the first 3 months poststroke.

## 1. Introduction

Stroke is one of the most common causes of mortality and long-term disability [1]. The risk of life-long stroke in adult women and men is approximately 25% [2]. Advances in acute stroke treatment have increased the survival rates after stroke. Patients with stroke need rehabilitation due to different rates of disability [3]. After treatment of acute stroke, physical rehabilitation is an important part of stroke management and is necessary to compensate for disabilities and to maximize functional performance. Many factors affect the success of rehabilitation treatment. Regardless of the cause of the stroke, factors such as patient age, stroke severity, comorbid diseases, degree of the deficit, and the nutritional status of the patient affect poststroke rehabilitation success.

Vitamin D deficiency is very common in Turkish society (73.9%) [4]. Vitamin D deficiency is a common problem in patients with stroke, and its prevalence in this patient group is about 71% [5]. Common causes of vitamin deficiency in patients with stroke are malnutrition, immobility, and insufficient sunlight exposure. Low serum vitamin D levels in these patients cause musculoskeletal problems and recent studies demonstrated that it also increased stroke severity, disability, cerebrovascular accidents, and cardiovascular death and mortality [6]. 

Vitamin D is very important for nervous system functions. It has a significant neuroprotective effect as a neurosteroid, and vitamin D receptors are widely expressed in neuronal and glial cells. Vitamin D increases neurotrophin production and secretion; it is involved in the synthesis of neuromediators and intracellular Ca homeostasis and prevention of oxidative damage in nerve tissue. In clinical studies, the frequency of some central nervous system diseases (schizophrenia and multiple sclerosis) has been shown to increase with vitamin D deficiency [7]. In a metaanalysis, it was reported that dementia is more common in vitamin D deficiency [8]. The correlation between neurodegenerative diseases and vitamin D deficiency may be related to the role of vitamin D in the regulation of nerve growth factor synthesis. Dysregulation of neuronal Ca levels negatively affects neuronal functions [9]. Vitamin D is important for the development and differentiation of neuronal cells. Vitamin D is also a micronutrient that acts as an antioxidant in the central nervous system. Calcitriol increases iNOS synthesis and the amount of glutathione in the central nervous system. Thus, it reduces oxidative stress and provides vasodilation [10]. Its role in Ca metabolism makes vitamin D important for the control of the relaxation response of striated muscle. In vitamin D deficiency, oxidative stress increases in striated muscle, and mitochondrial dysfunction increases and muscle atrophy may be observed [11]. Because of these effects of vitamin D on nervous and musculoskeletal systems, it is reasonable to expect adequate vitamin D levels and proper supplementation will have positive effects on poststroke rehabilitation.

In the literature, there are contradictory results in studies that investigated the effect of vitamin D supplementation on poststroke rehabilitation [12,13]. The aim of the current study was to compare the lower extremity motor function and ambulation gains obtained with poststroke rehabilitation in patients with stroke who received vitamin D supplementation. 

## 2. Materials and methods

The study was conducted retrospectively between September 2020 and October 2020 at Gazi University Faculty of Medicine Physical Medicine and Rehabilitation Department. The study protocol was approved by the Gazi University Faculty of Medicine ethics committee (Decision Number: 559). Seventy-six patients who received inpatient stroke rehabilitation treatment between May 2018 and February 2020 were included in the study. The demographic and clinical data of the patients were collected by reviewing electronic and physical patient files. Patients who had stroke for the first time were enrolled in the study. Physical examination and imaging methods [computed tomography (CT), magnetic resonance imaging (MRI)] were used for differential diagnosis. The exclusion criteria were: (1) absence of prerehabilitation vitamin D level measurement, (2) having chronic kidney, liver, or lung diseases that might interfere with vitamin D levels, (3) being on a current steroid treatment, and (4) previous history of orthopedic problems known to affect lower extremity functions. Patients’ age, sex, time elapsed from the onset of stroke to the start of rehabilitation, duration of rehabilitation, type of stroke, comorbid diseases, and nutritional status were recorded. 25-hydroxyvitamin D (25(OH)D) serum levels measured as ng/mL in the first week after hospitalization were recorded. Lower extremity motor function and ambulation were evaluated using Brunnstrom Recovery Stage (BRS) (lower extremity) and functional ambulation classification (FAC). The BRS assessment scores the clinical severity of hemiplegia from 1 to 6. A score of 1 indicates paralysis and 6 indicates normal force and function [14]. FAC evaluates ambulation in 6 categories ranging from 0 to 5. A score of 0 means that the patient is not ambulatory, and 5 indicates normal ambulation [15]. The patients included in the study were divided into two groups as those who received vitamin D treatment during the rehabilitation period and those who did not. Weekly vitamin D (50,000 IU) support for 4–12 weeks was given to patients orally during the rehabilitation period and the total vitamin D dose ranged from 200,000 to 600,000 IU. Vitamin D levels before rehabilitation, BRS and FAC scores, and changes in BRS and FAC scores before and after rehabilitation were compared between the two groups.

SPSS v 22 (IBM Corp., Armonk, NY, USA) data analysis program was used for statistical analysis. For comparison of demographic features, the Mann–Whitney U test was used for nonparametric continuous variables, and the chi-square test was used for discrete variables. In the presentation of statistical data, continuous variables are expressed as median, minimum–maximum values, and discrete variables as percentages. A p-value of less than 0.05 was considered to be statistically significant.

## 3. Results

There were 76 patients enrolled in the study. Thirty-seven (49%) of these patients did not receive vitamin D treatment during rehabilitation and 39 (51%) did. Some of the demographic characteristics of the patients are summarized in Table 1.

**Table 1:** Demographic data and clinical characteristics of the patients.

	Vitamin D		
	Control (n = 37)	Vitamin D (n = 39)	p–value
Age (years)	60 (14–89)	64 (25–83)	0.917
Sex			
Female	16 (21%)	26 (34%)	0.069
Male	21 (28%)	13 (17%)	
Stroke			
Hemorrhagic	10 (13%)	6 (8%)	0.365
Ischemic	23 (30%)	30 (39%)	
Tumor	4 (5%)	3 (4%)	
Stroke–rehabilitation interval (months)	4 (0.5–72)	3 (0.5–156)	0.975
Stroke			
Subacute (1–4 week)	12 (16%)	12 (16%)	0.876
Chronic (>4 week)	25 (33%)	27 (36%)	
Side			
Right	18 (24%)	19 (25%)	0.367
Left	19 (25%)	18 (24%)	
Bilateral	0	2 (2%)	
Previous rehab			
Yes	14 (18%)	11 (14%)	0.372
No	23 (30%)	28 (37%)	
Rehabilitation duration(days)	43 (22–90)	43 (22–90)	0.451

Prerehabilitation vitamin D levels of patients are summarized in Figure. There were four patients with normal vitamin D levels (5.3%). The median value of vitamin D levels of all patients before rehabilitation treatment was 17 ng/mL (8–41).

**Figure:**
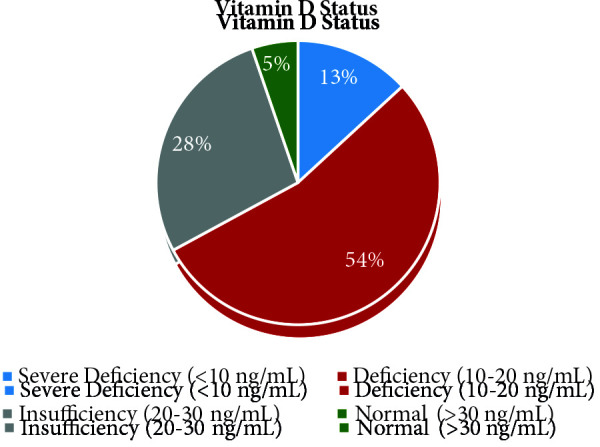
Distribution of patients’ vitamin D levels.

In 32% of patients (n = 24), the time elapsed after the stroke was less than 3 months. This period was more than 3 months in 68% (n = 52).

The comparison of FAC and BRS scores of patients who did and did not receive vitamin D treatment during the rehabilitation process is shown in Table 2. There was no statistically significant difference in the initial FAC and BRS scores between those who did and did not receive vitamin D treatment during rehabilitation (p = 0.872 and p = 0.906). Postrehabilitation FAC and BRS scores were also similar in both groups (p = 0.151 and p = 0.153). However, the change in FAC and BRS scores after rehabilitation treatment was higher in the group receiving vitamin D (p = 0.005 and p = 0.018).

**Table 2:** Effect of vitamin D treatment on ambulation and lower extremity motor function.

	Vitamin D		
	Control (n = 37)	Vitamin D (n = 39)	p-value
FAC			
Prerehab	3 (1–5)	2 (0–4)	0.872
Postrehab	3 (0–5)	3 (0–5)	0.151
Change	0 (0–3)	1 (0–4)	0.005
BRS			
Prerehab	2 (0–6)	4 (1–6)	0.906
Postrehab	4 (1–6)	5 (1–6)	0.153
Change	0 (0–2)	1 (0–5)	0.018
Vitamin D* levels (ng/mL)	18 (8–41)	15 (8–36)	0.330

Values are presented as median (min–max). FAC: functional ambulation classification, BRS: Brunnstrom recovery stage.

When the patients who received rehabilitation treatment for the first time were examined (n = 51), no difference was observed between the two groups in terms of pre- and postrehabilitation FAC and BRS scores (p > 0.05). However, the FAC and BRS score changes after rehabilitation treatment were statistically different between the two groups (p = 0.035 and p = 0.024). The improvement in these evaluations was higher in the group receiving vitamin D treatment. The initial vitamin D levels were similar in these patients (p = 0.543) [controls: 18 (8–41) ng/mL and Vitamin D: 15 (8–28) ng/mL] (Table 3).

**Table 3:** The effect of vitamin D therapy on ambulation and lower extremity motor function in patients undergoing rehabilitation for the first time.

	Vitamin D		
	Control (n = 23)	Vitamin D (n = 28)	p–value
FAC			
Prerehab	2 (0–5)	2 (0–4)	0.842
Postrehab	3 (0–5)	3 (0–5)	0.366
Change	0 (0–3)	1 (0–4)	0.035
BRS			
Prerehab	3 (1–6)	4 (1–6)	0.855
Postrehab	4 (1–6)	5 (1–6)	0.213
Change	0 (0–2)	1 (0–5)	0.024
Vitamin D* levels (ng/mL)	18 (8–41)	15 (8–36)	0.543

Values are presented as median (min–max). FAC: functional ambulation classification, BRS: Brunnstrom recovery stage.

In previously rehabilitated patients (n = 25), FAC and BRS scores before and after rehabilitation and changes in these scores were similar (p > 0.05). Initial vitamin D levels were also similar in these patients (p = 0.564) (Table 4).

**Table 4:** The effect of vitamin D treatment on ambulation and lower extremity motor function in patients who had received previous rehabilitation treatment.

	Vitamin D		
	Control (n = 14)	Vitamin D (n = 11)	p–value
FAC			
Prerehab	3 (0–4)	3 (0–4)	0.602
Postrehab	3 (2–4)	4 (2–5)	0.114
Change	0 (0–1)	1 (0–3)	0.176
BRS			
Prerehab	3 (2–6)	3 (2–5)	0.841
Postrehab	3 (2–6)	3 (2–6)	0.799
Change	0 (0–2)	0 (0–1)	0.507
Vitamin D* levels (ng/mL)	21 (11–36)	19 (8–36)	0.564

Values are presented as median (min–max). FAC: functional ambulation classification, BRS: Brunnstrom recovery stage.

The effects of vitamin D treatment on FAC and BRS scores were compared in the patients who started rehabilitation treatment in the first 3 months after stroke. The change in FAC and BRS scores was found to be statistically significant in patients receiving vitamin D treatment (p = 0.005 and p = 0.047) (Table 5).

**Table 5:** The effect of vitamin D treatment on ambulation and lower extremity motor function in patients with stroke in the first 3 months after stroke.

	Vitamin D		
	Control (n = 22)	Vitamin D (n = 16)	p-value
FAC			
Prerehab	1.5 (0–5)	1 (0–4)	0.795
Postrehab	2.5 (0–5)	4 (1–5)	0.251
Change	0 (0–3)	2 (0–5)	0.005
BRS			
Prerehab	3.5 (1–6)	3,5 (1–6)	0.978
Postrehab	4.5 (1–6)	5 (2–6)	0.171
Change	0 (0–2)	1 (0–5)	0.047
Vitamin D* levels (ng/mL)	17.5 (8–26)	13 (8–36)	0.200

Values are presented as median (min–max). FAC: functional ambulation classification, BRS:

In patients who were not within the first 3 months after stroke, vitamin D treatment had no effect on FAC and BRS scores (p > 0.05) (Table 6).

**Table 6:** Effect of vitamin D treatment on ambulation and lower extremity motor function in patients with stroke who were not within the first 3 months of stroke.

	Vitamin D		
	Control (n = 19)	Vitamin D (n = 19)	p-value
FAC			
Prerehab	3 (0–4)	3 (0–4)	0.692
Postrehab	3 (0–5)	3 (0–5)	0.441
Change	0 (0–3)	1 (0–3)	0.311
BRS			
Prerehab	3 (1–6)	4 (1–6)	0.999
Postrehab	4 (1–6)	5 (1–6)	0.580
Change	0 (0–2)	0 (0–3)	0.169
Vitamin D* levels (ng/mL)	20 (8–41)	19 (8–35)	0.759

Values are presented as median (min–max). FAC: Functional ambulation classification, BRS: Brunnstrom recovery stage.

## 4. Discussion

In this clinical trial, it was observed that vitamin D supplementation during stroke rehabilitation might have positive effects on ambulation and lower extremity motor functions. The positive change in FAC and BRS scores in patients receiving vitamin D treatment was found to be statistically significant (p > 0.05). There are studies in the literature investigating the effect of patient vitamin D levels on rehabilitation success. We think that this study is valuable in terms of investigating the effect of vitamin D supplementation given during rehabilitation.

The effect of vitamin D levels on the functional outcomes of rehabilitation has been studied in many different disease groups such as spinal cord injury, fibromyalgia, and stroke [16–18]. Liu et al. conducted a metaanalysis investigating the effect of serum vitamin D level on functional results in patients with stroke; they examined 10 studies including 6845 patients with stroke and concluded that vitamin D deficiency affected functional gains negatively [18]. There are also studies reporting that vitamin D deficiency is a predictor of overall prognosis in patients with stroke and increases morbidity and mortality [19–21]. On the other hand, there are contradictory results regarding the effect of vitamin D supplementation in the rehabilitation success of patients with stroke. In a randomized controlled trial, Momosaki et al. compared placebo with 2000 IU/day vitamin D treatment given during 8 weeks’ rehabilitation treatment of patients with acute stroke. Functional outcomes before and after rehabilitation were evaluated using the Barthel index and Brunnstrom motor recovery stage and it was reported that vitamin D supplementation did not improve functional gains [12]. However, a relatively low dose of vitamin D was used in this study. Therefore, adequate vitamin D levels may not have been reached and the desired effect may not have been observed. Gupta et al. evaluated the effect of high-dose vitamin D treatment (600,000 IU) on functional gains using modified Rankin scores in patients with acute stroke with low vitamin D levels and stated that functional gains were better in those receiving vitamin D support [13]. 

In the general population, it is recommended to provide weekly vitamin D (50,000 IU) support for 8–12 weeks in the treatment of severe vitamin D deficiency [22]. As far as we know, a special vitamin D treatment regimen recommended for patients with stroke with vitamin D deficiency is not available. Narasimhan et al. compared the rehabilitation success between patients receiving 600,000 IU cholecalciferol supplementation and patients without vitamin D supplementation using the Scandinavian stroke scale in patients with ischemic stroke. It was reported that there was a significant improvement in stroke outcomes after 3 months in patients who were supplemented with vitamin D [23]. Sarı et al. administered 300,000 IU cholecalciferol (IM) at the beginning of rehabilitation in chronic stroke patients and investigated its effect on rehabilitation outcomes using BRS, FAC, the modified Bartel index, and Berg balance scale (BBS) at the beginning of rehabilitation and 3rd month of vitamin D administration. It was reported that vitamin D treatment increased activity levels and accelerated balance recovery, but did not significantly affect ambulation or motor recovery [24]. The results obtained by Sarı et al. regarding ambulation and motor recovery are different from those obtained in our study. This may be due to the fact that the patients included in the study were not within the first 3 months after stroke and in our study, oral vitamin D treatment was given to patients during the rehabilitation period and the total vitamin D dose ranged from 200,000 to 600,000 IU. In our study, the changes in BRS and FAC scores evaluating functional activity in patients receiving vitamin D treatment were found to be better in the group of patients who were rehabilitated for the first time. The change in BRS and FAC scores was higher in patients who started rehabilitation in the first 3 months after stroke and received vitamin D treatment. This effect was not observed in those who had previously received rehabilitation treatment. There was no significant difference in BRS and FAC scores in patients who started rehabilitation after the 3rd month of stroke. Most of the functional motor gains in patients with stroke occur in the first 3 months after stroke [25]. For this reason, vitamin D treatment may not have contributed to the changes in BRS and FAC scores in patients within the chronic period and patients who had previously received rehabilitation. We think that vitamin D treatment is beneficial and important, especially in patients who are in the first 3 months after stroke and who are rehabilitated for the first time. 

The most important limitations of this study are its retrospective design and the fact that vitamin D supplementation has not been used in different treatment regimens and doses. We think that there is a need for randomized controlled studies investigating the effects of different doses of vitamin D treatment. Another limitation is that only the FAC and BRS stages are used as the outcome measures to assess ambulation and mobility. Due to the retrospective design of the study, scales such as Functional Independence Measure and BBS, which can provide information about general functional gains and balance, could not be used. Another limitation of the study is the lack of knowledge about whether patients with previous rehabilitation treatment were evaluated for vitamin D levels during their previous rehabilitation treatment and whether these patients received vitamin D support. One of the reasons for not observing the effect of vitamin D supplementation in these patients in the chronic phase may be due to the fact that the maximum recovery expected in these patients has already occurred in the first 3 months, which are critical. However, we think that it is important that vitamin D supplementation has a positive effect on BRS and FAC score changes in patients who are in the first 3 months after stroke and who have not received any rehabilitation before.

As a result, we think that vitamin D supplementation in patients with stroke may increase rehabilitation success, especially in patients who are in the first 3 months after stroke and who will receive rehabilitation treatment for the first time.

## Funding

The authors received no financial support for the research and/or authorship of this article.

## Informed consent

Study protocol received institutional review board approval and that all participants provided informed consent in the format required by the relevant authorities and/or boards. The study protocol was approved by the Gazi University Faculty of Medicine ethics committee (07/09/2020. Decision Number: 559).

## References

[ref1] (2013). Measuring the global burden of disease. New England Journal of Medicine.

[ref2] (2018). GBD 2016 Lifetime Risk of Stroke Collaborators. Global, regional, and country-specific lifetime risks of stroke, 1990 and 2016. New England Journal of Medicine.

[ref3] (2017). Stroke rehabilitation. CONTINUUM: Lifelong Learning in Neurology.

[ref4] (2017). and risk factors for vitamin D deficiency in patients with widespread musculoskeletal pain. Turkish Journal of Medical Sciences.

[ref5] (2014). Vitamin D deficiency and osteoporosis in stroke survivors: an analysis of National Health and Nutritional Examination Survey (NHANES). Journal of Vascular and Interventional Neurology.

[ref6] (2017). Decrement of serum vitamin D level after stroke. Annals of Rehabilitation Medicine.

[ref7] (2013). Vitamin D and the central nervous system. Pharmacological Reports.

[ref8] (2017). Vitamin D deficiency as a risk factor for dementia: a systematic review and meta-analysis. BMC Geriatrics.

[ref9] (2018). The role of vitamin D in brain health: a mini literature review. Cureus.

[ref10] (1998). Expression of inducible nitric oxide synthase during rat brain inflammation: regulation by 1. Glia.

[ref11] (2019). Mechanisms of vitamin D on skeletal muscle function: oxidative stress, energy metabolism and anabolic state. European Journal of Applied Physiology.

[ref12] (2019). D supplementation and post-stroke rehabilitation: a randomized, double-blind, placebo-controlled trial. Nutrients.

[ref13] (2016). Effect of Vitamin D and calcium supplementation on ischaemic stroke outcome: a randomised controlled open‐label trial. International Journal of Clinical Practice.

[ref14] (1966). Motor testing procedures in hemiplegia: based on sequential recovery stages. Physical Therapy.

[ref15] (1986). Gait assessment for neurologically impaired patients: standards for outcome assessment.

[ref16] (2011). An effective oral vitamin D replacement therapy in persons with spinal cord injury. The Journal of Spinal Cord Medicine.

[ref17] (2018). Vitamin D supplementation seems to improve fibromyalgia symptoms: Preliminary results. The Israel Medical Association Journal.

[ref18] (2019). Prognostic utility of serum 25-hydroxyvitamin D in patients with stroke: A meta-analysis. Journal of Neurology.

[ref19] (2016). Serum 25‐hydroxyvitamin D deficiency predicts poor outcome amongst acute ischaemic stroke patients with low high density lipoprotein cholesterol. European Journal of Neurology.

[ref20] (2014). Association between serum concentration of vitamin D and 1-year mortality in stroke patients. Cerebrovascular Diseases.

[ref21] (2015). Serum vitamin D status as a predictor of prognosis in patients with acute ischemic stroke. Cerebrovascular Diseases.

[ref22] (2010). Vitamin D deficiency in adults: when to test and how to treat. In Mayo Clinic Proceedings.

[ref23] (2017). Role of vitamin D in the outcome of ischemic stroke-a randomized controlled trial. Journal of Clinical and Diagnostic Research.

[ref24] (2018). A randomized, double-blind study to assess if vitamin D treatment affects the outcomes of rehabilitation and balance in hemiplegic patients. Journal of Physical Therapy Science.

[ref25] (2017). Spontaneous and therapeutic-induced mechanisms of functional recovery after stroke. Translational Stroke Research.

